# Protective Effect of Carvacrol against Gut Dysbiosis and *Clostridium difficile* Associated Disease in a Mouse Model

**DOI:** 10.3389/fmicb.2017.00625

**Published:** 2017-04-21

**Authors:** Shankumar Mooyottu, Genevieve Flock, Abhinav Upadhyay, Indu Upadhyaya, Kendra Maas, Kumar Venkitanarayanan

**Affiliations:** ^1^Department of Animal Science, University of ConnecticutStorrs, CT, USA; ^2^Microbial Analysis, Resources, and Services, University of ConnecticutStorrs, CT, USA

**Keywords:** *Clostridium difficile*, carvacrol, microbiome, gut dysbiosis, mouse model

## Abstract

This study investigated the effect of carvacrol (CR), a phytophenolic compound on antibiotic-associated gut dysbiosis and *C. difficile* infection in a mouse model. Five to six-week-old C57BL/6 mice were randomly divided into seven treatment groups (challenge and control) of eight mice each. Mice were fed with irradiated feed supplemented with CR (0, 0.05, and 0.1%); the challenge groups were made susceptible to *C. difficile* by orally administering an antibiotic cocktail in water and an intra-peritoneal injection of clindamycin. Both challenge and control groups were infected with 10^5^CFU/ml of hypervirulent *C. difficile* (ATCC 1870) spores or PBS, and observed for clinical signs for 10 days. Respective control groups for CR, antibiotics, and their combination were included for investigating their effect on mouse enteric microflora. Mouse body weight and clinical and diarrhea scores were recorded daily post infection. Fecal samples were collected for microbiome analysis using rRNA sequencing in MiSeq platform. Carvacrol supplementation significantly reduced the incidence of diarrhea and improved the clinical and diarrhea scores in mice (*p* < 0.05). Microbiome analysis revealed a significant increase in Proteobacteria and reduction in the abundance of protective bacterial flora in antibiotic-treated and *C. difficile*-infected mice compared to controls (*p* < 0.05). However, CR supplementation positively altered the microbiome composition, as revealed by an increased abundance of beneficial bacteria, including Firmicutes, and significantly reduced the proportion of detrimental flora such as Proteobacteria, without significantly affecting the gut microbiome diversity compared to control. Results suggest that CR could potentially be used to control gut dysbiosis and reduce *C. difficile* infection.

## Introduction

*Clostridium difficile* infection is the major cause of antibiotic-associated diarrhea in hospital settings around the world (McFarland, 2008; Hookman and Barkin, 2009). *C. difficile* principally causes a serious toxin-mediated colitis in the elderly and immunocompromised patients (Weese, 2010). Annually, more than 300,000 cases of *C. difficile* associated diseases (CDAD) are reported in the United States, resulting in more than US$3 billion as health care costs (Wilkins and Lyerly, 2003; Ghose et al., 2007). A recently emerged, highly toxigenic and hyper-virulent *C. difficile* strain NAP1/ribotype 027 has been implicated in increasing incidence of CDAD among patients all over the world (Sunenshine and McDonald, 2006; Blossom and McDonald, 2007; Hookman and Barkin, 2009).

*C. difficile* infection has been associated with the use of antibiotics and gastric acid suppressing agents that result in gut dysbiosis (Bartlett, 1992; Kelly and LaMont, 1998; Dial et al., 2005). Prolonged antibiotic therapy results in the disruption of the normal enteric microflora, leading to an altered microbial composition such as increased population of Proteobacteria and reduced proportion of Bacteroides and Firmicutes in the gut microbiome (Shahinas et al., 2012; Ling et al., 2014; Seekatz and Young, 2014; Theriot et al., 2014). Consequently, gut dysbiosis results in the germination of spores and selection for *C. difficile* in the intestine. Following spore germination and outgrowth in the presence of a disrupted gut flora, the vegetative cells of *C. difficile* produce potent toxins known as toxin A and toxin B (Voth and Ballard, 2005). *Clostridium difficile* toxins (A and B) are functionally glucosyl transferases, which inactivate the Rho family GTPases associated with F-actin regulation, and cause disruption of the cytoskeleton and intestinal epithelial tight junctions (von Eichel-Streiber et al., 1999; Keel and Songer, 2006). This leads to a severe inflammatory response with the release of cytokines and leukotrienes, causing pseudomembrane formation and severe diarrhea (McDonald et al., 2006; Sunenshine and McDonald, 2006; Hookman and Barkin, 2009). Since gut dysbiosis is considered as the most important predisposing factor in CDAD, emerging and novel therapeutic approaches, including fecal microbiome transplantation (FMT) primarily aimed at restoration of the normal gut flora in CDAD patients are explored (Kassam et al., 2013).

Despite the fact that a majority of the currently used antibiotics can predispose CDAD by disrupting the normal gut flora, antibiotics are still used as the primary line of treatment against infection (Bartlett, 1992; O'Connor et al., 2004). Nonetheless, many of the anti- *C. difficile* antibiotics are found to predispose CDAD in patients by inducing gut dysbiosis (O'Connor et al., 2004; McFarland, 2008; Shah et al., 2010). Moreover, the emergence of antibiotic resistant strains of hypervirulent *C. difficile* is documented worldwide (Spigaglia et al., 2011; Steiner et al., 2012). The Centers for Disease Control and Prevention (CDC) recently listed *C. difficile* as one among the three urgent threats in their report on emerging pathogens with antibiotic resistance (Steiner et al., 2012). Since the toxins are the major virulence factors for CDAD, a search for alternative therapeutic agents, which can reduce *C. difficile* virulence without affecting normal gastrointestinal flora opens a new research area.

Carvacrol (CR) is a food grade, monoterpenoid phytophenol that is naturally present in oregano and thyme oil. Diverse pharmacological actions of carvacrol, including antimicrobial and anti-inflammatory activities have been previously demonstrated (Baser, 2008). A recent study from our laboratory suggested the potential use of CR as an anti-*C. difficile* therapeutic agent due to its inhibitory effect on *C. difficile* toxin production without affecting the growth of beneficial gut bacteria *in vitro* (Mooyottu et al., 2014a). This study demonstrated that CR significantly inhibited toxin production in hypervirulent *C. difficile* strains by modulating toxin production genes. Therefore, this study investigated the therapeutic effect of CR against *C. difficile* in an *in vivo* model, specifically its impact on the clinical course of *C. difficile* infection and the host microbiome. Mouse is a well-established model of *C. difficile* infection (Chen et al., 2008; Sun et al., 2011), and antibiotic-associated *C. difficile* infection can be induced in a mouse model by administering antibiotics orally and intraperitoneally, followed by inoculation of *C. difficile* spores (Chen et al., 2008; Sun et al., 2011).

## Materials and methods

### Ethics statement, animals, and housing

This study was conducted with the approval of the Institutional Animal Care and Use Committee (IACUC) of the University of Connecticut. All recommended guidelines for the care and use of animals were followed. Six-week-old C57BL/6 mice were obtained from Charles River (Boston, MA). Animals were housed in a biohazard level II, AALAC-accredited facility and monitored twice daily for health. Mice were provided with autoclaved food, water, and bedding, with 12-h light/dark cycles. All cage changes, *C. difficile* spore infection, and sample collections were performed under a laminar flow hood using properpersonal protective equipment. The work area was sterilized using 10% bleach between experimental treatment groups to prevent cross-contamination. The mice were housed in pairs in a cage, and four cages were included for each treatment in each of the experiments.

### Mouse model of *C. difficile* infection and treatment groups

The infection model adopted for this study is a modification of the method described by Chen et al. (2008). Five to six-week-old female mice were randomly divided into eight treatment groups of eight animals each (Table 1). The animals were subjected to food restriction for 12 h, and given powdered feed supplemented with 0, 0.05, and 0.10% of CR. After 7 days, an antibiotic mixture was added in drinking water (kanamycin,0.4 mg/mL, gentamicin,0.03 mg/mL, colistin,850 U/mL, metronidazole, 0.215 mg/mL, and vancomycin,0.045 mg/mL) for 3 days [challenge groups (Ant + CD, Ant + CD + CR 0.05%, and Ant + CD + CR 0.1%), and the antibiotic control group (Ant control)]. After the antibiotic treatment, the mice were given regular autoclaved water for 2 days, and all animals in the challenge groups (Ant + CD, Ant + CD + CR 0.05%, and Ant + CD + CR 0.1%), and the antibiotic control group (Ant control) received a single dose of clindamycin (10 mg/kg, maximum volume of injection 0.5 ml/mouse using a 27 gauge gavage needle and syringe) intraperitoneally 1 day before *C difficile* challenge. This antibiotic pre-treatment was intended to disrupt the normal gut flora of mice and facilitate *C. difficile* colonization. All animals in the challenge groups (Ant + CD, Ant + CD + CR 0.05%, and Ant + CD + CR 0.1%) were infected by oral gavage with 10^5^ colony-forming units (CFU) per 0.1 ml total volume of hypervirulent *C. difficile* spores (ATCC BAA 1805) using a straight 18-gauge needle with 1” shaft length, and were monitored for signs of CDAD such as diarrhea, hunched posture and wet tail using a mouse clinical score sheet (Supplementary Table 1). Animals were observed twice daily for 10 days for mortality and morbidity. The individual weight of each animal was measured every day. Fecal samples from all animals were collected on alternate days post infection. The animals were euthanized at the end of the experiment (10th day after *C. difficile* challenge).

**Table 1:** **Different treatment groups used in the experiment**.

**Treatment group (*n* = 8 each)**	**Antibiotic**	**CR**	***C. difficile***
Negative control	−	−	−
Ant + CD	+	−	+
Ant + CR	+	0.1%	−
CR control	−	0.1%	−
Ant + CD + CR 0.05%	+	0.05%	+
Ant + CD + CR 0.1%	+	0.1%	+
Ant control	+	−	−

*Ant, Antibiotic; CD, C. difficile; CR, Carvacrol*.

### DNA extraction, PCR amplification, and sequencing of taxonomic marker

DNA was extracted from 0.25 g of fecal sample (collected on the 2nd day post inoculation, DPI) from all treatment groups using the MoBio PowerMag Soil 96 well kit (MoBio Laboratories, Inc), according to the manufacturer's protocol for the Eppendorf ep Motion liquid handling robot. DNAquantification was performed using the Quant-iT PicoGreen kit (Invitrogen, ThermoFisher Scientific). Partial bacterial 16S rRNA genes (V4) were amplified using 30 ng extracted DNA as template. The V4 region was amplified using 515F and 806R with Illumina adapters and dual indices (8 basepair golay on 3′; Caporaso et al., 2012), and eight basepair on the 5′ (Kozich at al., 2013). Samples were amplified in triplicate using Phusion High-Fidelity PCR master mix (New England BioLabs) with the addition of 10 μg BSA (New England BioLabs). The PCR reaction was incubated at 95°C for 3.5 min, then 30 cycles of 30 s at 95.0°C, 30 s at 50.0°C, and 90 s at 72.0°C, followed by a final extension at 72.0°C for 1 min were used. The PCR products were pooled for quantification and visualization using the QIAxcel DNA Fast Analysis (Qiagen). The PCR products were normalized based on the concentration of DNA from 250 to 400 bp and pooled using the QIAgility liquid handling robot. The pooled PCR products were cleaned using the Gene Read Size Selection kit (Qiagen) according to the manufacturer's protocol. The cleaned pool was sequenced on MiSeq using v2 2 × 250 base pair kit (Illumina, Inc).

### Sequence analysis

The microbiome analysis was set up as a completely randomized design with treatments done in replicates of six. The sequences were filtered and clustered using Mothur 1.36.1 based on a published protocol with slight modifications (Kozich et al., 2013). Operational taxonomic units (OTUs) were clustered at 97% sequence similarity. Downstream analysis of samples was done using R version 3.2. The alpha diversity was calculated using inverse Simpson to measure the richness and evenness of the OTUs. The effect of treatment on the alpha diversity was analyzed using Tukey's Test. A permutational multivariate analysis (PERMANOVA, adonis function, 75 permutations) was conducted to analyze the effect of various treatments on the bacterial community composition. Significant change in alpha diversity was determined by Anova followed by Tukey's honest significant differences adjusting for multiple comparisons (*p* = 0.05). NMS ordinations were run in R (v 3.3.0) using metaMDS in the vegan (v 2.3-5) package after calculating the stress scree plots to determine the number of axes required to achieve stress below 0.2, plotted using ggplot2 (v 2.1.0). Finally, the relative abundance of OTUs of major phyla, order, and genera was determined to assess the effect of treatment. Tukey's Test was used to identify changes in groups of bacteria based on treatment and the significance was detected at *P* < 0.05.

### Statistical analysis

The results were expressed as means ± standard errors of the means (SEM). The differences between the experimental groups were compared using the analysis of variance (ANOVA). Two-way ANOVA was used to compare experimental groups across the days. The differences between two groups were analyzed using unpaired Student's *t*-test. “N1” Chi-squared test were used to compare incidence rate between two different treatments. The statistical significance level was set at a *P* < 0.05.

## Results

### Effect of CR supplementation on the incidence of diarrhea and severity of *C. difficile* infection in mice

**Figure 1:**
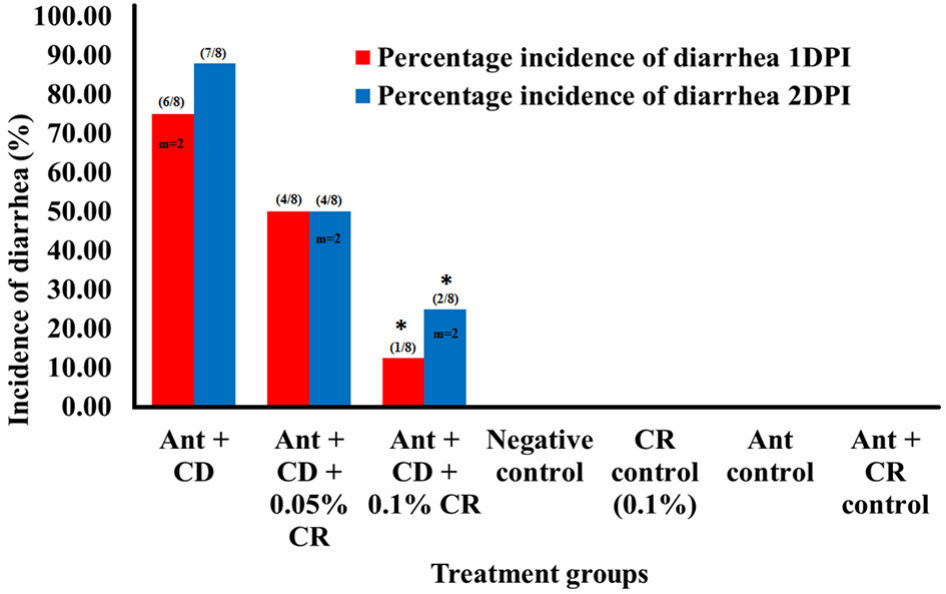
**Effect of CR supplementation on the incidence of ***C. difficile*** associated diarrhea in mice**. The incidence of diarrhea in different treatement groups was recorded after *C. difficile* challenge. Groups: (1) Negative Control: Mice treated with no CR, no antibiotics and no *C. difficile* (2) CR control: Mice fed with 0.1% CR in feed, (3) Ant Control: Mice administered with antibiotic cocktail in water and an intra-peritoneal injection of clindamycin, (4) Ant + CR Control: Mice fed with CR (0.1%) supplemented feed and administered with antibiotic cocktail in water and an intra-peritoneal injection of clindamycin, (5) Ant + CD: administered with antibiotic cocktail in water and an intra-peritoneal injection of clindamycin, and infected by *C. difficile* (6) (Ant + CD + 0.05% CR): Mice fed with CR (0.05%), administered with antibiotic cocktail in water and an intra-peritoneal injection of clindamycin, and infected with *C. difficile*) (Ant + CD + 0.1% CR): Mice fed with CR (0.1%), administered with antibiotic cocktail in water and an intra-peritoneal injection of clindamycin, and infected with *C. difficile*. (^*^ treatments significantly differed from infected control group (Ant + CD) *p* < 0.05; m indicates the number of mortalities recorded; cumulative incidence of diarrhea per total number of animals used in the experiment is shown in parenthesis).

In order to assess the prophylactic effect of CR against *C. difficile* associated diarrhea in mice (8 mice per treatment group), the animal diets were supplemented with CR at two different concentrations (0.05% and 0.1%) in feed prior to antibiotic treatment and subsequent *C. difficile* infection (Ant + CD + 0.05% CR and Ant + CD + 0.1% CRThe oral administration of 10^5^ CFU/ml NAP1 *C. difficile* spores resulted in high morbidity and low mortality in infected mice. In *C. difficile* infected control groups (Ant + CD), 75% (6/8) of the animals showed severe diarrhea on 1DPI, and 90% (7/8) of the animals showed severe diarrhea on 2DPI. Two animals died on 1DPI, and no further mortality was observed in this group. No increase in the incidence of diarrhea was observed after 2DPI (Figure 1). Interestingly, only 50% (4/8) of the animals showed diarrhea in 1DPI in Ant + CD + 0.05% CR group (animals supplemented with 0.05% CR prior to the antibiotic treatment and subsequent *C. difficile* infection), no increase in the incidence of diarrhea was reported after 1DPI. In Ant + CD + 0.1% CR group, only 12.5% (1/8) of the animals showed diarrhea on 1DPI, which further increased to 25% (2/8) on 2DPI and no further incidents were observed on subsequent days. In both CR-treated and *C. difficile* infected groups (Ant + CD + 0.05% CR and Ant + CD + 0.1% CR), two mortalities each were recorded on 2DPI. No diarrheal symptoms were observed in control groups (Negative control, CR control, Ant control, and Ant + CR control).

### Effect of CR supplementation on clinical score and body weight of *C. difficile* infected mice

**Figure 2:**
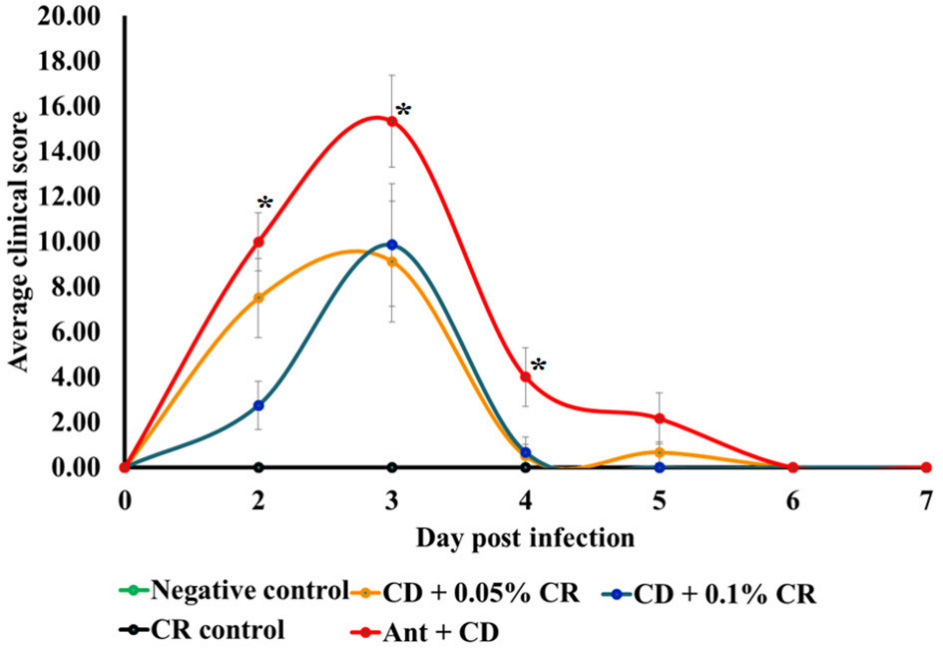
**Effect of CR supplementation on the severity of ***C. difficile*** associated disease in mice**. The severeity of *C. difficile* associated disease in different treatment groups was determined based on a clinical score sheet. Groups: (1) Negative Control: Mice treated with no CR, no antibiotics and no *C. difficile* (2) CR control: Mice fed with 0.1% CR in feed, (3) Ant Control: Mice administered with antibiotic cocktail in water and an intra-peritoneal injection of clindamycin, (4) Ant + CR Control: Mice fed with CR (0.1%) supplemented feed and administered with antibiotic cocktail in water and an intra-peritoneal injection of clindamycin, (5) Ant + CD: administered with antibiotic cocktail in water and an intra-peritoneal injection of clindamycin, and infected by *C. difficile* 6) (Ant + CD + 0.05% CR): Mice fed with CR (0.05%), administered with antibiotic cocktail in water and an intra-peritoneal injection of clindamycin, and infected with *C. difficile*) (Ant + CD + 0.1% CR): Mice fed with CR (0.1%), administered with antibiotic cocktail in water and an intra-peritoneal injection of clindamycin, and infected with *C. difficile*. (^*^The clinincal scores of positive control group (Ant + CD were significantly greater than that of Ant + CD + CR 0.1% and control groups, *p* < 0.05).

Clinical scores of the individual animals in different groups were recorded using standard clinical score chart, from 1DPI to 7 DPI (Chen et al., 2008; Sun et al., 2011). The *C. difficile* control group (Ant + CD group) exhibited significantly increased severity, as indicated by a higher average clinical score per group on 1, 2, 3, and 4 DPI (Figure 2) (*p* < 0.05). The severity of CDAD in animals supplemented prophylactically with 0.05 and 0.1% CR (Ant + CD + 0.05% CR and Ant + CD + 0.1% CR groups) was lesser than that of the untreated group (Ant + CD) (*p* < 0.05). On 1DPI, there was a dose-dependent reduction in the severity of infection in CR supplemented groups. Irrespective of the treatments, all surviving morbid animals recovered by 6DPI, as indicated by a zero clinical score.

**Figure 3:**
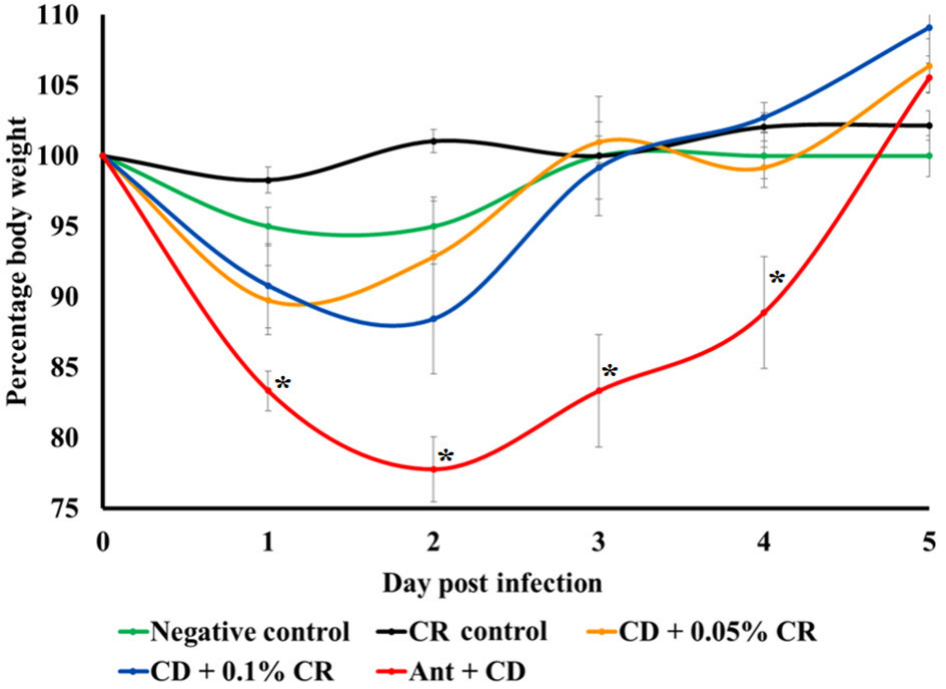
**Effect of CR supplementation on relative weight loss in ***C. difficile*** infected and non-infected mice**. The body weights of the animals were recorded daily and the relative percentage weight with respect to the initial weight prior to the infection was calculated. Groups: (1) Negative Control: Mice treated with no CR, no antibiotics and no *C. difficile* (2) CR control: Mice fed with 0.1% CR in feed, (3) Ant Control: Mice administered with antibiotic cocktail in water and an intra-peritoneal injection of clindamycin, (4) Ant + CR Control: Mice fed with CR (0.1%) supplemented feed and administered with antibiotic cocktail in water and an intra-peritoneal injection of clindamycin, (5) Ant + CD: administered with antibiotic cocktail in water and an intra-peritoneal injection of clindamycin, and infected by *C. difficile* (6) (Ant + CD + 0.05% CR): Mice fed with CR (0.05%), administered with antibiotic cocktail in water and an intra-peritoneal injection of clindamycin, and infected with *C. difficile*) (Ant + CD + 0.1% CR): Mice fed with CR (0.1%), administered with antibiotic cocktail in water and an intra-peritoneal injection of clindamycin, and infected with *C. difficile*. (^*^The relative weight loss of positive control group (Ant + CR) was significantly greater than Ant + CD + CR 0.05%, Ant + CD + CR 0.1% and control groups, *p* < 0.05).

A similar trend was observed in average body weights of animals in different treatment groups. Body weights were recorded daily and the relative percentage weight with respect to the initial weight before *C. difficile* infection was calculated (Figure 3). Carvacrol alone (CR control) and in combination with antibiotic (Ant + CR control) did not cause any significant weight loss compared to the negative control. All mice in the *C. difficile* control group (Ant + CD) showed significant and progressive weight loss from 1 DPI to 5DPI compared to the negative control (*p* < 0.05). However, CR-treated and *C. difficile* infected mice (Ant + CD + 0.05% CR and Ant + CD + 0.1% CR groups) showed a significantly lesser weight loss in comparison to untreated and *C. difficile* infected group (Ant + CD) from 1 DPI and 2DPI, with regaining of the initial weight on 3DPI (*p* < 0.05). No significant difference in recorded weight loss was observed between 0.05 and 0.1% CR-treated *C. difficile* infected mice except for a rapid and early increase in the body weight on 2DPI in the 0.05% CR group.

### Effect of CR supplementation on the gut microbiome of *C. difficile* infected and non-infected mice

**Figure 4:**
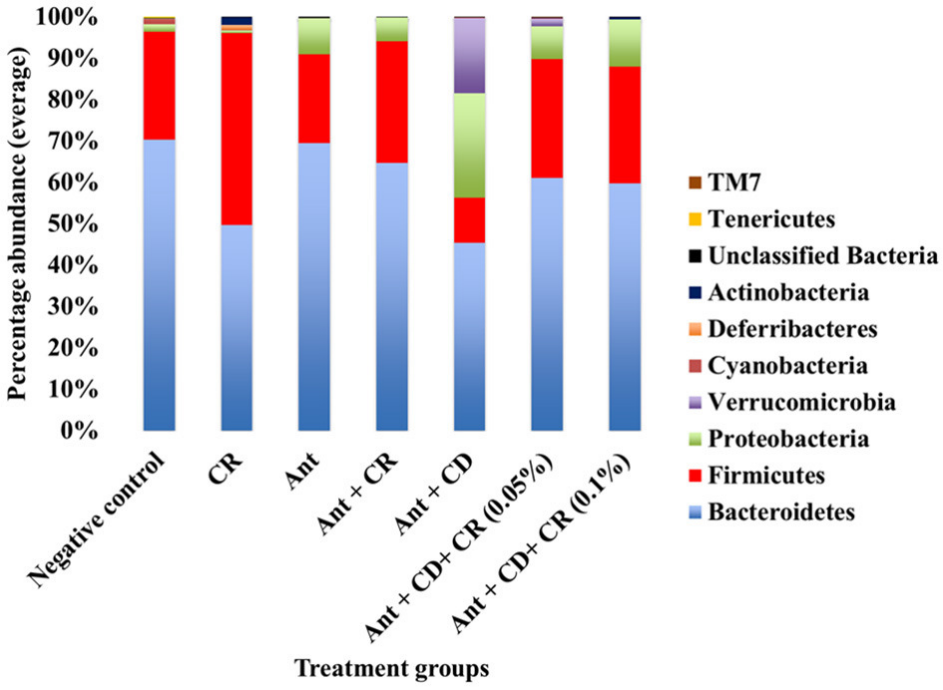
**Effect of CR supplementation on the abundance of major gut microbiota (phyla level) in the antibiotic treated and ***C. difficile*** challenged mice**. The relative abundance of OTUs at different taxonomic levels was determined by gut microbiome analysis. Groups: (1) Negative Control: Mice treated with no CR, no antibiotics and no *C. difficile* (2) CR control: Mice fed with 0.1% CR in feed, (3) Ant Control: Mice administered with antibiotic cocktail in water and an intra-peritoneal injection of clindamycin, (4) Ant + CR Control: Mice fed with CR (0.1%) supplemented feed and administered with antibiotic cocktail in water and an intra-peritoneal injection of clindamycin, (5) Ant + CD: administered with antibiotic cocktail in water and an intra-peritoneal injection of clindamycin, and infected by *C. difficile* (6) (Ant + CD + 0.05% CR): Mice fed with CR (0.05%), administered with antibiotic cocktail in water and an intra-peritoneal injection of clindamycin, and infected with *C. difficile*) (Ant + CD + 0.1% CR): Mice fed with CR (0.1%), administered with antibiotic cocktail in water and an intra-peritoneal injection of clindamycin, and infected with *C. difficile*.

Microbiome analysis results revealed specific patterns in the composition of different bacterial taxa in different treatment groups. In the phylum level, the gut microbiome of negative control mice was predominated by Bacteriodetes, followed by Firmicutes (Figure 4) with a minimal proportion of other phyla, including Proteobacteria. A similar trend was observed in CR control group, with an abundance of Bacteriodetes followed by Firmicutes, although the proportion of Firmicutes was slightly higher than that of the negative control group. Antibiotic administration significantly increased the proportion of Proteobacteria in antibiotic-treated (Ant control) group compared to the negative control and CR control groups (*p* < 0.05). Interestingly, supplementation of CR along with antibiotic (Ant + CR control) significantly reduced the abundance of Proteobacteria compared to the antibiotic only (Ant control) group. The *C. difficile* control group (Ant + CD), where *C. difficile* spores were orally gavaged after antibiotic treatment, exhibited a remarkably increased abundance of Proteobacteria along with a greater proportion of Verrucomicrobia compared to all other control groups (Negative control, CR control, Ant control, Ant + CR control groups) (*p* < 0.05). In addition, the abundance of Bacteriodetes and Firmicutes was significantly reduced in *C. difficle* control group (Ant + CD), compared to uninfected controls (*p* < 0.05). Strikingly, this alteration in the abundance of Proteobacteria, Firmicutes, Bacteriodetes and Verrucobacteria due to *C. difficile* infection was reversed significantly by CR supplementation, as observed in the CR-treated and *C. difficile* infected groups (Ant + CD + 0.05% CR and Ant + CD + 0.10% CR groups) (*p* < 0.05).

**Figure 5:**
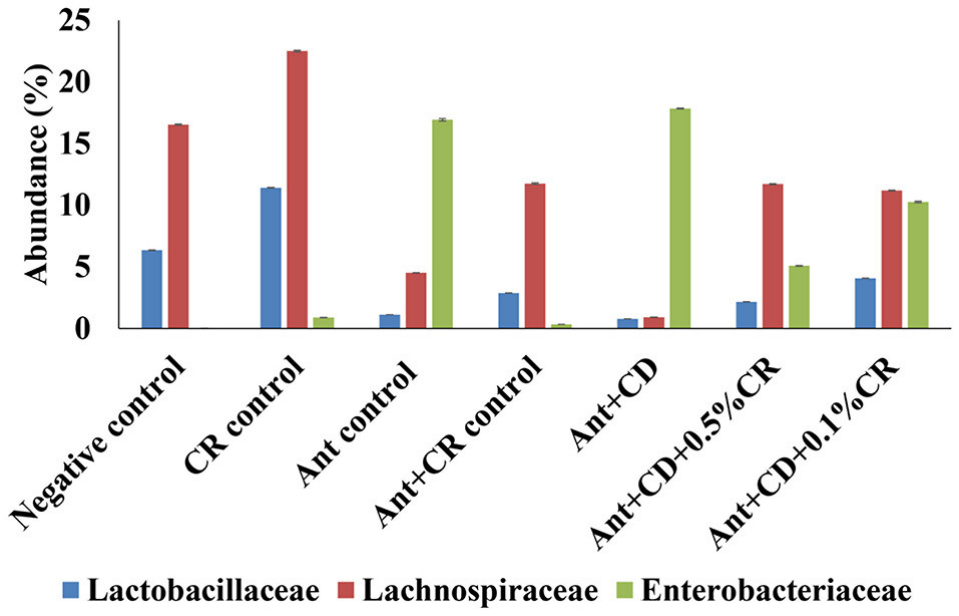
**Effect of CR supplementation on the abundance of Enterobacteriaceae, Lactobacillaceae and Lachnospiraceae in the antibiotic treated and ***C. difficile*** challenged mice**. The relative abundance of OTUs at family level (Family. Enterobacteriaceae, Lactobacillaceae and Lachnospraceae) was by determined using gut microbiome analysis. Groups: (1) Negative Control: Mice treated with no CR, no antibiotics and no *C. difficile* (2) CR control: Mice fed with 0.1% CR in feed, (3) Ant Control: Mice administered with antibiotic cocktail in water and an intra-peritoneal injection of clindamycin, (4) Ant + CR Control: Mice fed with CR (0.1%) supplemented feed and administered with antibiotic cocktail in water and an intra-peritoneal injection of clindamycin, (5) Ant + CD: administered with antibiotic cocktail in water and an intra-peritoneal injection of clindamycin, and infected by *C. difficile* (6) (Ant + CD + 0.05% CR): Mice fed with CR (0.05%), administered with antibiotic cocktail in water and an intra-peritoneal injection of clindamycin, and infected with *C. difficile* (7) (Ant + CD + 0.1% CR): Mice fed with CR (0.1%), administered with antibiotic cocktail in water and an intra-peritoneal injection of clindamycin, and infected with *C. difficile*. (Treatments significantly differed from respective control groups, *p* < 0.05).

At the Order and Family level, an increased abundance of Enterobacteriaceae was observed in antibiotic alone (Ant control) and *C. difficile* (Ant + CD) groups compared to negative control and CR control groups (Figure 5). Moreover, CR supplementation significantly reduced the abundance of Enterobacteriaceae induced by the antibiotic administration and *C. difficile* infection, as indicated by a significant reduction in their abundance in Ant + CR control group, Ant + CD + 0.05% CR, and Ant + CD + 0.10% CR groups (*p* < 0.05) (Figure 5). Carvacrol treatment significantly increased the abundance of Lactobacillaceae and Lachnospiraceae in the gut microbiome compared to that of negative control (Figure 5) (*p* < 0.05). The abundance of Lactobacillaceae and Lachnospiraceae was significantly reduced following antibiotic treatment (Ant control group) and *C. difficile* (Ant + CD control group) infection, compared to the Negative control and CR alone (CR control) groups (*p* < 0.05). This effect was significantly reversed by the supplementation of CR in Ant + CR control, Ant + CD + 0.5 CR, and Ant + CD + 0.5 CR groups.

**Figure 6:**
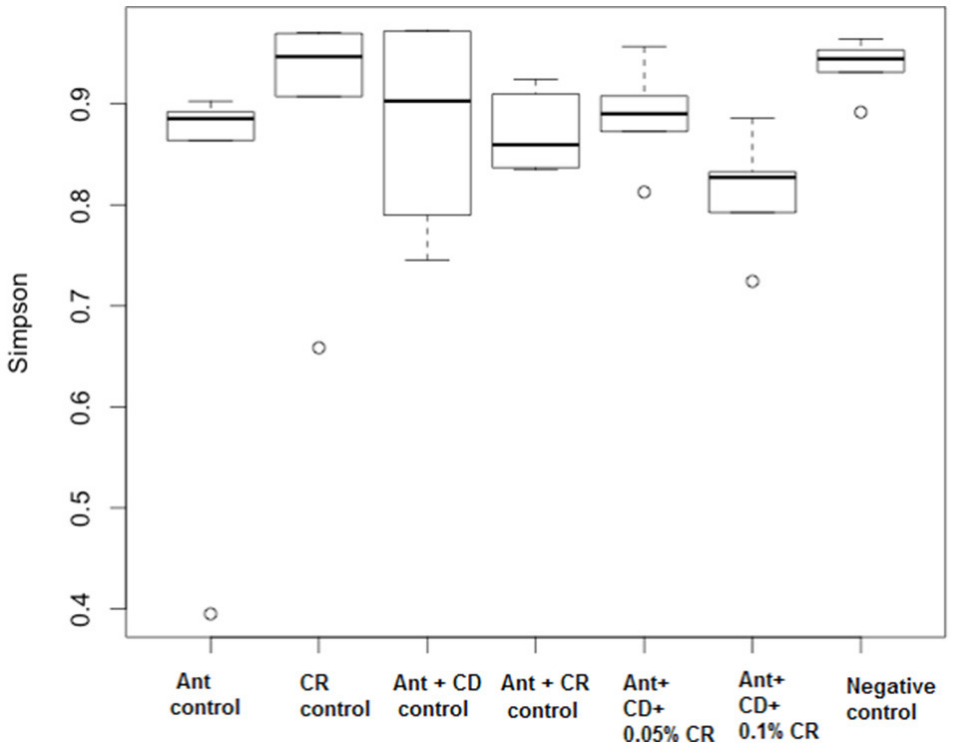
**Effect of CR supplementation on the diversity of gut microbiota of antibiotic treated and ***C. difficile*** challenged mice**. Alpha diversity of the gut microbiome was calculated by using inverse Simpson to measure the richness and evenness of the OTUs. Groups: (1) Negative Control: Mice treated with no CR, no antibiotics and no *C. difficile* (2) CR control: Mice fed with 0.1% CR in feed, (3) Ant Control: Mice administered with antibiotic cocktail in water and an intra-peritoneal injection of clindamycin, (4) Ant + CR Control: Mice fed with CR (0.1%) supplemented feed and administered with antibiotic cocktail in water and an intra-peritoneal injection of clindamycin, (5) Ant + CD: administered with antibiotic cocktail in water and an intra-peritoneal injection of clindamycin, and infected by *C. difficile* (6) (Ant + CD + 0.05% CR): Mice fed with CR (0.05%), administered with antibiotic cocktail in water and an intra-peritoneal injection of clindamycin, and infected with *C. difficile* (7) (Ant + CD + 0.1% CR): Mice fed with CR (0.1%), administered with antibiotic cocktail in water and an intra-peritoneal injection of clindamycin, and infected with *C. difficile*.

**Figure 7:**
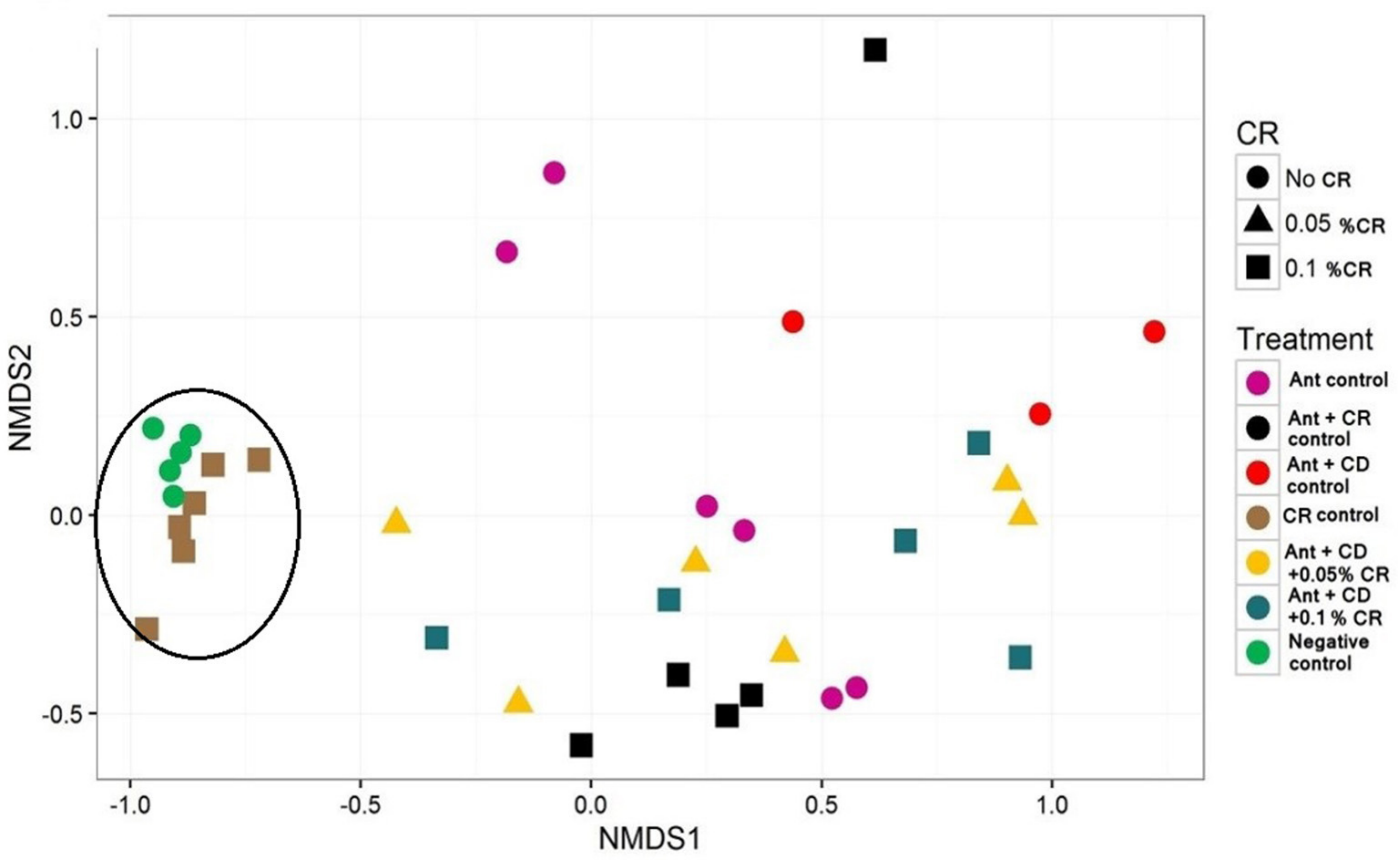
**Effect of CR supplementation on the diversity of gut microbiota of antibiotic treated and ***C. difficile*** challenged mice**. Relationships between treatment groups based on the abundance of species present in each sample were plotted. Groups: (1) Negative Control: Mice treated with no CR, no antibiotics and no *C. difficile* (2) CR Control: Mice fed with 0.1% CR in feed, (3) Ant Control: Mice administered with antibiotic cocktail in water and an intra-peritoneal injection of clindamycin, (4) Ant + CR Control: Mice fed with CR (0.1%) supplemented feed and administered with antibiotic cocktail in water and an intra-peritoneal injection of clindamycin, (5) Ant + CD: administered with antibiotic cocktail in water and an intra-peritoneal injection of clindamycin, and infected by *C. difficile* (6) (Ant + CD + 0.05% CR): Mice fed with CR (0.05%), administered with antibiotic cocktail in water and an intra-peritoneal injection of clindamycin, and infected with *C. difficile* (7) (Ant + CD + 0.1% CR): Mice fed with CR (0.1%), administered with antibiotic cocktail in water and an intra-peritoneal injection of clindamycin, and infected with *C. difficile*. Circle indicates close clustering of CR Control samples and Nagative Control samples.

Inverse Simpson plot revealed a differential pattern of bacterial diversity in various treatment groups (Figure 6). Strikingly, CR treatment did not alter the diversity of the gut bacterial community compared to the untreated control group (Negative control). As expected, antibiotic treatment significantly reduced the bacterial diversity compared to control and CR group. There was a marked reduction in the diversity of the bacterial community in *C. difficile* infected groups, irrespective of the CR treatment. Moreover, NMDS plot representing the relationships between samples in various treatment groups based on the abundance of species present in each sample revealed a close clustering of CR control samples and untreated control samples (Figure 7). This representation suggests that the species abundance in CR treatment groups is comparable to untreated mice indicating minimal effect of CR on gut microbial diversity.

## Discussion

Prolonged antibiotic therapy and subsequent gut dysbiosis result in *C. difficile* spore germination, and colonization of the large intestine with vegetative cells of the bacterium, and subsequent production of toxins TcdA and TcdB, resulting in *C. difficile* associated diarrhea (Hookman and Barkin, 2009). *C. difficile* toxins lead to intestinal inflammation, increased epithelial permeability (Castagliuolo et al., 1998; Feltis et al., 2000; He et al., 2002), enhanced cytokine and chemokine production (Castagliuolo et al., 1998; He et al., 2002), neutrophil infiltration (Kelly et al., 1994) and the release of reactive oxygen intermediates (He et al., 2002), thereby causing direct damage to the intestinal mucosa (Ng et al., 2010). Antibiotics are the primary line of treatment in *C. difficile* infection, although the use of antibiotics has been documented for inducing and aggravating gut dysbiosis and relapse of the infection post-therapy. In addition, increasing incidence of *C. difficile* acquiring antibiotic resistance is reported worldwide.

In the current study, we investigated the prophylactic efficacy of CR as an alternative antimicrobial agent that can ameliorate *C. difficile* associated diarrhea without inducing gut dysbiosis. Previous studies have reported that supplementation of low doses of CR exerted no detrimental effects on endogenous bacterial populations, including *Lactobacilli* and *Bifidobacteria* in pigs and poultry (Jamroz et al., 2005; Si et al., 2006).

Previous studies conducted in our laboratory revealed that SICs of CR reduced *C. difficile* toxin production and cytotoxicity in Vero cells *in vitro* (Mooyottu et al., 2014a). In addition, our previous experiments showed an inhibition of *C. difficile* spore outgrowth in the presence of CR (Mooyottu et al., 2014b). The results from the mice experiment indicated that our *in vitro* results apparently well translated *in vivo* with regards to the clinical outcome and gut microbiome of the animals when prophylactically treated with CR prior to *C. difficile* infection. As expected, CR supplementation significantly reduced the incidence of diarrhea in *C. difficile* infected mice (*p* < 0.05). Moreover, CR supplementation significantly reduced the severity of clinical infection in *C. difficile* infected mice, as evident from a reduced average clinical score compared to the infected control group (Ant + CD) (*p* < 0.05). However, CR-treated and *C. difficile* infected mice exhibited significantly lesser weight loss compared to the untreated group (Ant + CD). The reduced severity of CDAD in CR-treated mice compared to infected control group (Ant + CD) could be attributed to an inhibitory effect of the phytochemical on *C. difficile* spore outgrowth and/or the toxin production, as observed in our *in vitro* studies. Moreover, CR has been reported to possess anti-inflammatory and anti-diarrheal properties (Baser, 2008), which could also have exerted a beneficial effect in *C. difficile* infected mice.

A healthy and normal gut microflora is crucial for preventing pathogen colonization and a variety of enteric bacterial infections, including *C. difficile* (Britton and Young, 2014). The most important predisposing factor for *C. difficile* infection is the disruption of normal gut microbiota (Hookman and Barkin, 2009). Antibiotic therapy significantly alters the microbial composition and diversity; and in many cases alterations in the microbial diversity persist to an extent, even after withdrawing antibiotic administration (Dethlefsen et al., 2008; Antonopoulos et al., 2009). In human patients, as age advances, the protective bacterial population of Firmicutes considerably diminishes accompanied by an increase in Bacteroidetes and undesirable species of Proteobacteria in the gut (Hopkins et al., 2001; Biagi et al., 2010; Claesson et al., 2011). Moreover, age-related senescence in the immune status of the elderly, along with frequent hospital visits during old age contribute to a detrimental alteration in the gut microbiome and subsequent colonization of *C. difficile* (Seekatz and Young, 2014). Other important factors that detrimentally affect the gut microbiota and predispose *C. difficile* infection are the use of proton pump inhibitors and chronic gastrointestinal diseases (Dial et al., 2005; Vesper et al., 2009; Berg et al., 2013). Proton pump inhibitors alter the pH of the gut, thereby affecting the microbial population, especially beneficial bacteria such as Lactobacillus species (Altman et al., 2008; Vesper et al., 2009). In addition, disease conditions such as inflammatory bowel disease (IBD) induce significant gut dysbiosis, which reduces the diversity of the protective population of Firmicutes and Bacteroides population accompanied by an increased Proteobacteria in the gut of affected patients (Manichanh et al., 2006; Nagalingam and Lynch, 2012). Increased abundance of Verrucomicrobia has also been shown in patients with antibiotic-associated gut dysbiosis (Weingarden et al., 2014). Moreover, the paucity of Firmicutes, especially the depletion of Ruminococcaceae, Lachnospiraceae, and butyrogenic bacteria within this phylum observed in *C. difficile* infection and nosocomial diarrhea in humans (Antharam et al., 2013). Moreover, *C. difficile* infected patients have a higher count of Enterobacteriaceae (Proteobacteria) and decreased Enterococcaceae (Firmicutes) (Hopkins and Macfarlane, 2002; Rea et al., 2012; Schubert et al., 2014).

The changes in the gut microbiome diversity and alterations in the relative abundance of different bacterial communities in human patients are replicated in mice models of *C. difficile* infection (Semenyuk et al., 2015). Antibiotic treatment and subsequent *C. difficile* infection significantly reduced the abundance of Firmicutes and Bacteroides in phylum level. Similar trends are observed in all taxonomical level such as a reduction in protective Lactobacillaceae, Lachnospiracea, and Bifidobacteriacea. A dramatic increase in the abundance of Proteobacteria specifically Enterobacteriaceae has been found in the antibiotic treated and *C. difficile* infected mice (Semenyuk et al., 2015). Similarly, antibiotic treatment and subsequent C. *difficile* infection significantly reduced microbiome diversity in mouse gut (Semenyuk et al., 2015).

In this study, CR treatment did not reduce the bacterial diversity in the mouse gut. To date, a majority of the antimicrobial compounds, especially antibiotics, have significantly altered the microbial diversity, and cause dysbiosis by changing the abundance of bacterial communities (Semenyuk et al., 2015). Moreover, CR treatment significantly increased the abundance of beneficial bacterial populations such as Firmicutes, specifically the members of Lactobacillaceae and Lachnospiraceae. In addition, CR treatment alone did not increase the abundance of detrimental bacterial populations compared to untreated control animals. Strikingly, CR reduced antibiotic-induced increases in the abundance of unfavorable bacterial populations such as Proteobacteria, specifically pathogenic gamma proteobacteria, including Enterobacteriaceae and other bacterial populations such as Verrucobacteria (Figure 5). Surprisingly, this beneficial shift brought about by CR treatment in the gut microbiome of antibiotic-treated and *C. difficile* infected animals is very much similar to that of human patients who have undergone fecal microbiome transplantation (Weingarden et al., 2014), which is documented as one of the most effective strategies against severe *C. difficile* infection (Schenck et al., 2015; Ofosu, 2016). These results suggest that reduced or delayed clinical infection rate and less severe clinical presentation of CR-treated animals could attributed in part to the beneficial shift in the gut microbiome.

To conclude, our results suggest CR supplementation to be protective against *C. difficile* infection in mice. Carvacrol supplementation significantly reduced the incidence of diarrhea and mitigated the severity of *C. difficile* induced clinical symptoms, inducing a favorable shift in the composition of the gut microbiota without detrimentally affecting the gut microbiome diversity in mice. These findings suggest the potential of CR as an anti- *C. difficile* agent, however, further clinical studies are warranted to confirm this.

## Author contributions

KV conceived the idea, KV and SM designed the experiments and prepared the manuscripts, SM, GF, and AU performed the experiments, KM and IU analyzed the data.

### Conflict of interest statement

The authors declare that the research was conducted in the absence of any commercial or financial relationships that could be construed as a potential conflict of interest.
