# Supplementary material for: Protective Effect of Carvacrol against Gut Dysbiosis and Clostridium difficile Associated Disease in a Mouse Model
Source: Front Microbiol. 2017 Apr 21;8:625. doi: 10.3389/fmicb.2017.00625 (PMC5399026; doi:10.3389/fmicb.2017.00625)
Supplement: Supplementary file 1 [file Table1.DOCX]

**Supplementary Table 1A: Mouse clinical score sheet:**

| **MOUSE CLINICAL SCORE SHEET** | | | | |
| --- | --- | --- | --- | --- |
|  | 0 | 1 | 3 | 5 |
| Coat | Normal | Lack of grooming | Rough/Ruffled fur |  |
| Activity | Normal | Isolated, abnormal posture | Huddled/inactive | Moribund,  unresponsive or seizuring |
| Breathing | Normal | Rapid, shallow | Rapid, abdominal | Laboured, blue |
| Movement | Normal | Slight incoordination/ abnormality | Incoordinated, walking of toes, reluctant to move | Staggering, paralysis, lack of mobility |
| Condition | BC 3 | BC 2+ | BC 2 | BC 1 |
| Dehydration | Nil | Skin less elastic | Skin tents | Skin tents, eyes sunken |
| Feces | Normal | Moist faeces | Diarrhoea or dry faeces | Uncontrolled diarrhoea, Wet tail |
| Body Weight | Normal | Markedly reduced growth (e.g. severe runting)  5% over 24 hours | Weight loss  up to 10% over 24 hours | Weight loss >10% over 24 hours or >20% over time |
| - Moribund animals (A lack of responsiveness to manual stimulation, lack of mobility or inability or failure to eat or drink or a clinical score > 25) will be euthanized. | | | | |

**Supplementary Table 1B. Mouse body condition chart**
